# Axial Spondyloarthritis and Autosomal Dominant Polycystic Kidney Disease in Two Siblings: A Rare Cooccurrence

**DOI:** 10.1155/2018/6150875

**Published:** 2018-03-04

**Authors:** Ozan Volkan Yurdakul, Abdulkerim Furkan Tamer, Okan Küçükakkaş, Aylin Rezvani

**Affiliations:** ^1^Department of Physical Medicine and Rehabilitation, Bezmialem Foundation University, Istanbul, Turkey; ^2^Department of Nephrology, Gaziosmanpasa Taksim Training and Research Hospital, Istanbul, Turkey

## Abstract

Autosomal dominant polycystic kidney disease (ADPKD) is the most frequently occurring hereditary kidney disease, and axial spondyloarthritis (SpA) is one of the most frequently occurring rheumatic diseases. Treatment-related decisions for axial SpA may pose a challenge in case of renal involvement. The authors describe two siblings with cooccurrence of these two diseases. The association of these two diseases is not well known. Practitioners should monitor renal function in SpA patients and take treatment-related decisions regarding renal involvement. Antitumor necrosis factor-alpha (anti-TNF-*α*) agents may be used in case nonsteroidal anti-inflammatory drugs (NSAIDs) cannot be utilized.

## 1. Introduction

Autosomal dominant polycystic kidney disease (ADPKD), the most common hereditary kidney disease, is characterized by impaired urinary-concentrating ability, hypertension, polyuria, nocturia, nephrolithiasis, hematuria, infections, and progressive loss of kidney function with a prevalence of 2.41–3.89/10000 [1, 2].

ADPKD manifestation is caused either by polycystin-1 (PC-1), encoded by *PKD1* located on chromosome 16p13 in 80%–85% of patients, or by polycystin-2 (PC-2), encoded by *PKD2* located on chromosome 4q21 in 10%–15% of patients [3, 4]. Defects in any of these genes cause abnormal primary ciliary function resulting in cyst formation and growth [5].

Spondyloarthritis is a group of chronic inflammatory diseases primarily affecting the axial skeleton with 0.5%–1.9% prevalence, making it one of the most common rheumatic diseases [6]. Ankylosing spondylitis, the prototype of axial spondyloarthritis, is a polygenic multifactorial disease, and human leukocyte antigen- (HLA-) B27 plays a causal role in its pathogenesis [7]. Axial spondyloarthritis (SpA) treatment includes physical exercise, nonsteroidal anti-inflammatory drugs (NSAIDs), sulphasalazine for peripheral arthritis, and antitumor necrosis factor-alpha (anti-TNF-*α*) in case NSAIDs are ineffective [8]. Renal involvement is a rare but important complication that is mostly discounted in young patients. Renal complications are estimated to occur in 8%–13.3% of patients with ankylosing spondylitis (AS) [9, 10]. Microscopic hematuria, proteinuria, increased serum creatinine, and nephrotic syndrome are major manifestations, and secondary amyloid A amyloidosis (62%) and immunoglobulin A (IgA) nephropathy (30%) are the two most frequently reported causes of renal involvement [11, 12]. Tubulointerstitial nephritis caused by NSAID use is another cause [8]; renal involvement varies from asymptomatic deterioration of renal function to end-stage renal failure [8].

Although there are numerous articles regarding the general causes of renal involvement in axial SpA in the literature, to our knowledge, association of ADPKD and axial SpA has never been reported. Here, we report two siblings with concurrent ADPKD and axial SpA.

## 2. Case Report

### 2.1. Case 1

**Figure 1:**
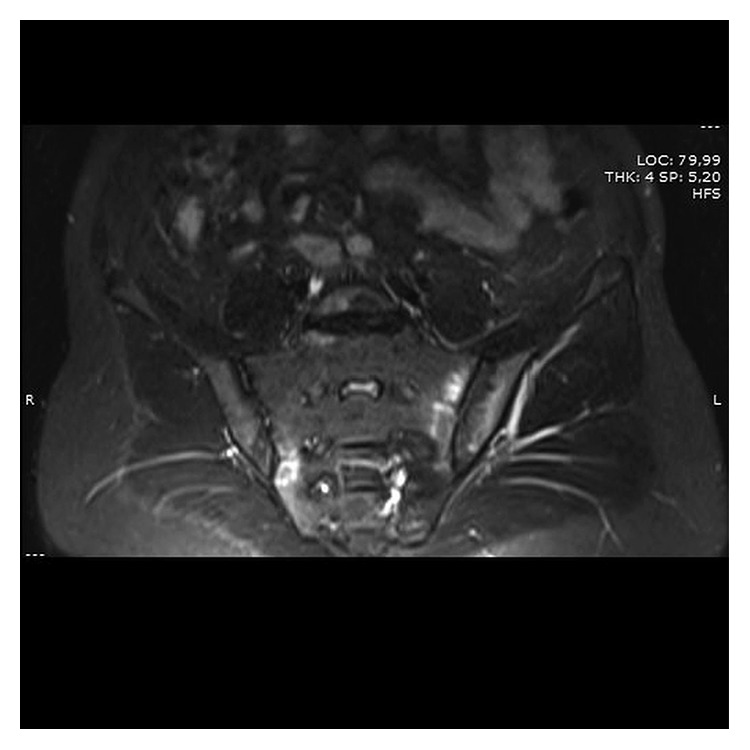
Coronal gadolinium-enhanced fat-suppressed T1-weighted MRI scan of the sacroiliac joints showing chronic degeneration and acute sacroiliitis.

**Figure 2:**
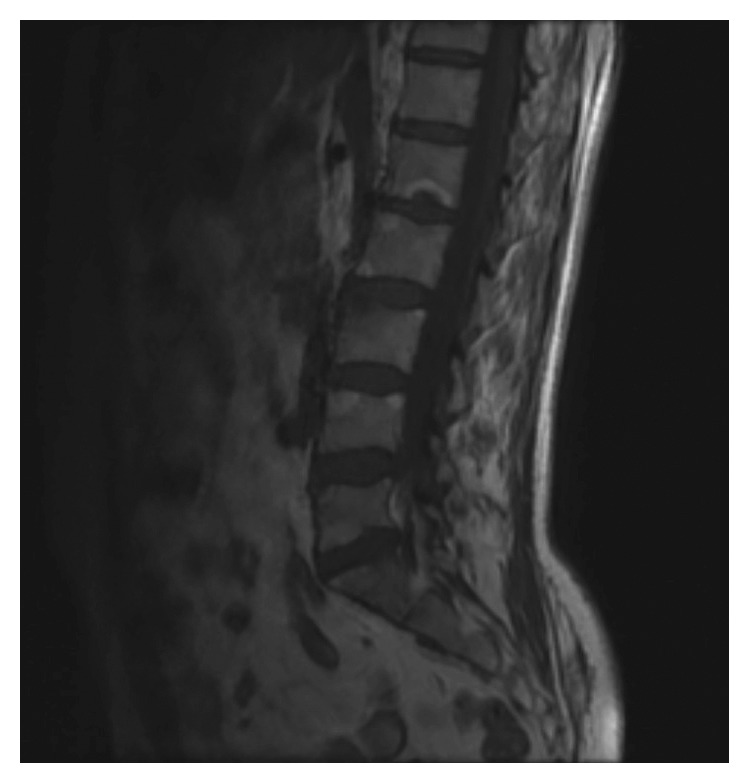
T1-weighted sagittal MRI scan of the lumbar spine indicating old Romanus lesions.

**Figure 3:**
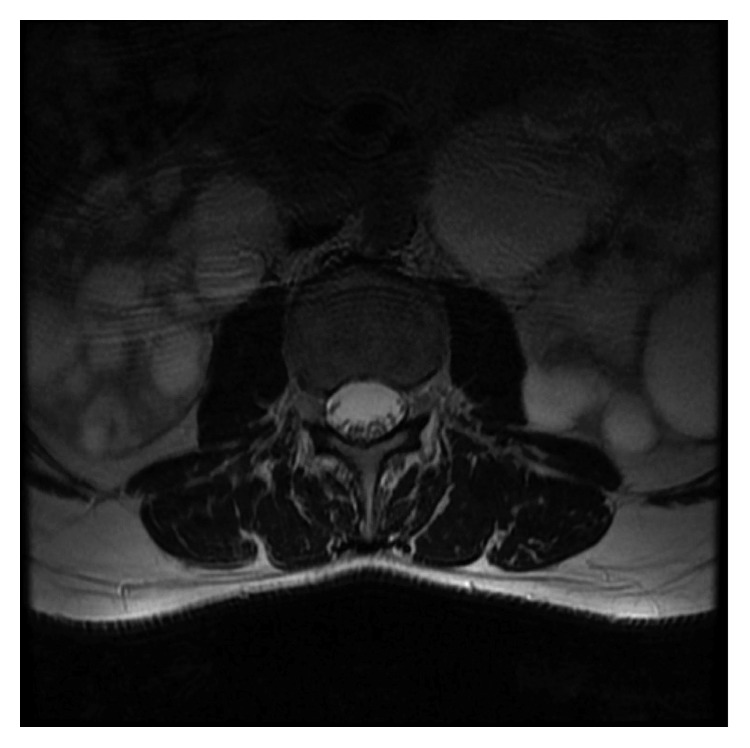
T2-weighted axial MRI scan indicating enlarged kidneys and multiple cysts.

A 37-year-old male was admitted to the outpatient clinic with back pain lasting for 10 years. He was diagnosed with axial SpA at another center 1 year before and treated with indomethacin 50 mg daily and sulphasalazine 2000 mg daily. He had 20 minutes of morning stiffness and augmented back pain while resting which was indicative of inflammatory back pain. He did not have history of inflammatory bowel diseases, and his examination did not indicate enthesitis or arthritis. His complete blood count was 6,700 cells/*µ*L white blood cells (WBCs) (N: 4,100–11,000), 4,240 cells/*µ*L neutrophils (N: 2,000–8,000), 13.02 g/dL hemoglobin (Hgb) (N: 11–18), 41.3% hematocrit (HCT) (N: 35–55), and 146,000 platelets (PLTs) (N: 150,000–400,000). C-reactive protein (CRP) was 6.71 mg/dL (N ≤ 3.48), erythrocyte sedimentation rate (ESR) 8 mm/h (N: 0–20), parathyroid hormone (PTH) 36.80 pg/ml (N: 12–88), serum creatinine (sCR) 0.81 mg/dL (N: 0.67–1.17), serum urea 46 mg/dL (N: 17–43), aspartate transaminase (AST) 16.9 U/L (N: 0–50), and alanine transaminase (ALT) 19 U/L (N: 0–50). Total urinalysis findings showed that urine density was 1.011 (N: 1.015–1.020) and other parameters were normal. The Rose Bengal test, hepatitis B surface antigen (HBs-Ag), anti-hepatitis C virus (HCV), and anti-Human Immunodeficiency Virus (HIV) were negative, and the patient was not HLA-B27 positive. A T1-weighted fat-suppressed gadolinium-enhanced MRI of the sacroiliac joints indicated joint space narrowing, bone erosions, subchondral sclerosis, and cortical irregularities in joint margins, indicating chronic damage and contrast agent uptake, both intra-articular and in the subchondral bone marrow, which is favorable for acute sacroiliitis (Figure 1). Lumbar MRI showed fatty degeneration on the anterior vertebral margins indicative of old Romanus lesions as well as multiple cysts and enlarged kidneys (Figures 2 and 3). These findings confirmed axial SpA diagnosis according to the Assessment of SpondyloArthritis international Society (ASAS) axial spondyloarthropathy classification criteria published in 2009 [13]. The clinical features, laboratory findings, and imaging results of the patient are shown in Table 1.

Following consultation with a nephrologist, the patient was diagnosed with ADPKD considering the existing family history of polycystic kidney disease, being asymptomatic until the 4th decade of life and occurring as multiple cysts and enlarged kidneys on MRI. The diagnosis of the ADPKD is based upon family history, clinical features, and imaging [4]. The patient was advised to use NSAIDs with caution as well as with frequent monitoring. Bath Ankylosing Spondylitis Disease Activity Index (BASDAI) was 6.5 and Bath Ankylosing Spondylitis Functional Index (BASFI) was 1.9. His indomethacin dose increased to 75 mg/day, and he was scheduled for a visit after 1 month, when his WBC was 6,300 cells/*µ*L, serum urea 40 mg/dL, uric acid 5.72 mg/dL (N: 3.5–7.2), sCR 0.77 mg/dL, CRP 4.01 mg/dL, and ESR 6 mm/h; total urinalysis showed that density was 1.013 and other parameters were normal; BASDAI was 4.7 and BASFI was 1.5. Hence, his serum CRP levels decreased and activity score improved; considering the patient's renal risk, we maintained daily indomethacin dose and scheduled him for follow-up. Five months later, BASDAI was 6.7 and BASFI was 2 with 1 hour of morning stiffness. Test results indicated that WBC was 6,300 cells/*µ*L, serum urea 29 mg/dL, uric acid 5.62 mg/dL, sCR 0.78 mg/dL, AST 17.4 U/L, ALT 14 U/L, CRP 10.9 mg/dL, and ESR 30 mm/h. Spot urine sample demonstrated a protein-to-creatinine ratio of 0.180. The patient was administered 50 mg/week etanercept due to disease activity deterioration and serum inflammatory markers. Three weeks after the initial etanercept administration, BASDAI was 4.3, BASFI was 1.5, and morning stiffness duration was 30 minutes. Test results showed that WBC was 5,600/*µ*L, serum urea 30 mg/dL, uric acid 6.37 mg/dL, sCR 0.82 mg/dL, serum sodium 138 mmol/L (N: 135–145), serum potassium 3.81 mmol/L (N: 3.5–5.1), AST 14.4 U/L, ALT 11 U/L, CRP 3.11 mg/dL, and ESR 7 mm/h. Total urinalysis findings demonstrated trace proteinuria on strip analysis. Spot urine sample demonstrated a protein-to-creatinine ratio of 0.133. The patient did not experience any side effects of etanercept regarding renal function besides a slight serum uric acid level increase.

One year after initial etanercept administration, the patient had BASDAI of 3.7 and BASFI of 1.4 with 20 minutes of morning stiffness. His final results were WBC 5,700 cells/*µ*L, Hgb 14.1 g/dL, PLT 210,000/*µ*L, serum urea 41 mg/dL, sCR 0.88 mg/dL, ALT 8 U/L, CRP 3.14 mg/dL, and ESR 9 mm/h. His final total urinalysis was normal, and uric acid levels slightly increased at follow-up with a final level of 6.8 mg/dL.

### 2.2. Case 2

**Figure 4:**
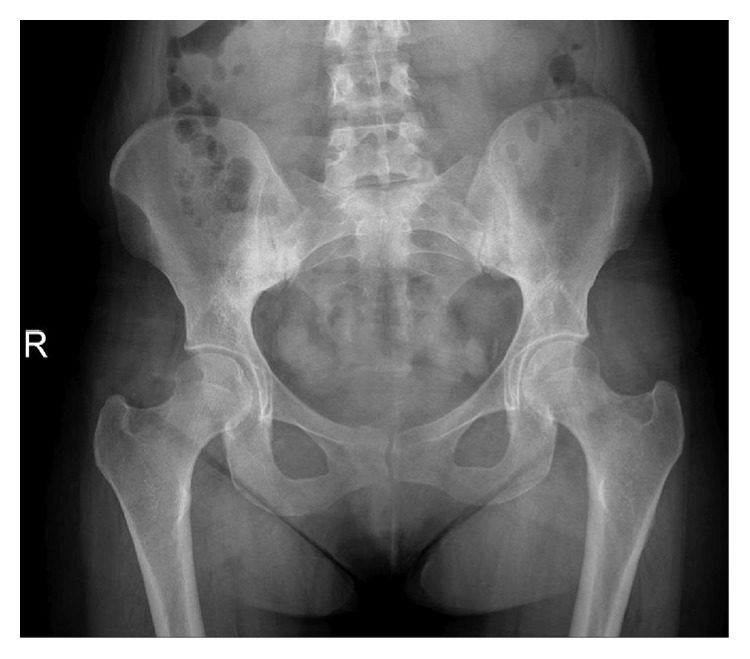
Plain radiograph of the sacroiliac joints demonstrating right grade 3 and left grade 2 sacroiliitis.

**Figure 5:**
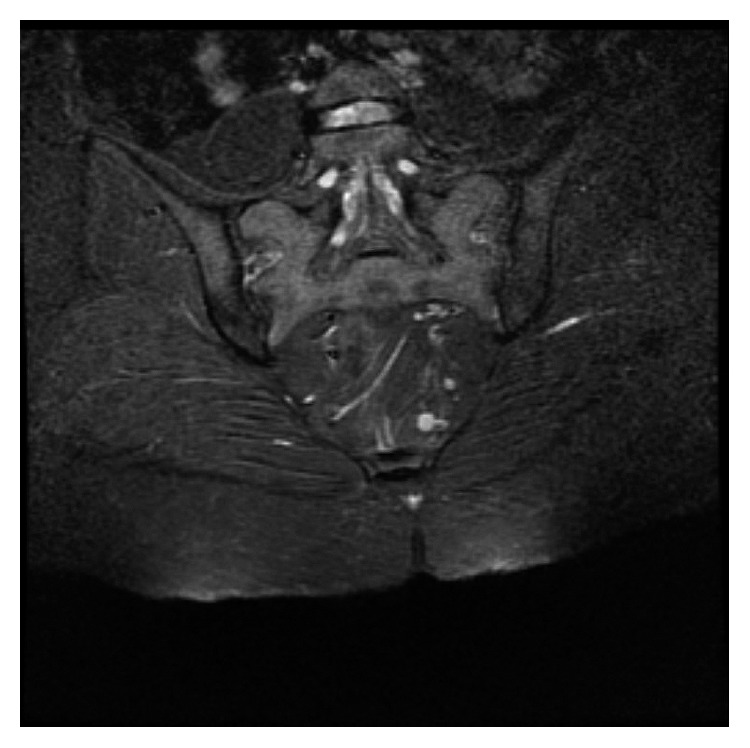
Coronal fat-suppressed T2-weighted MRI scan of the sacroiliac joints in favor of acute sacroiliitis and bilateral sclerosis.

**Figure 6:**
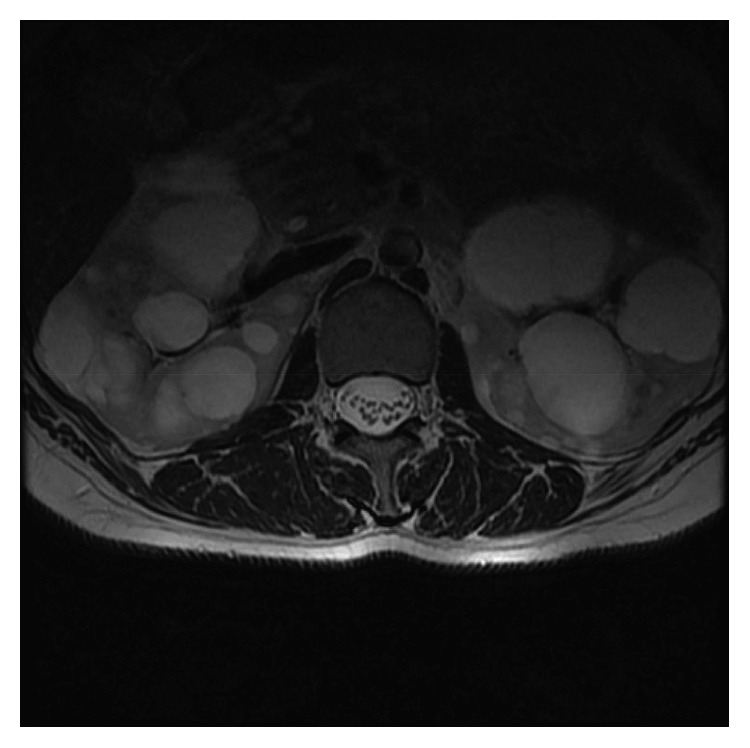
T2-weighted axial MRI scan showing polycystic kidneys.

**Figure 7:**
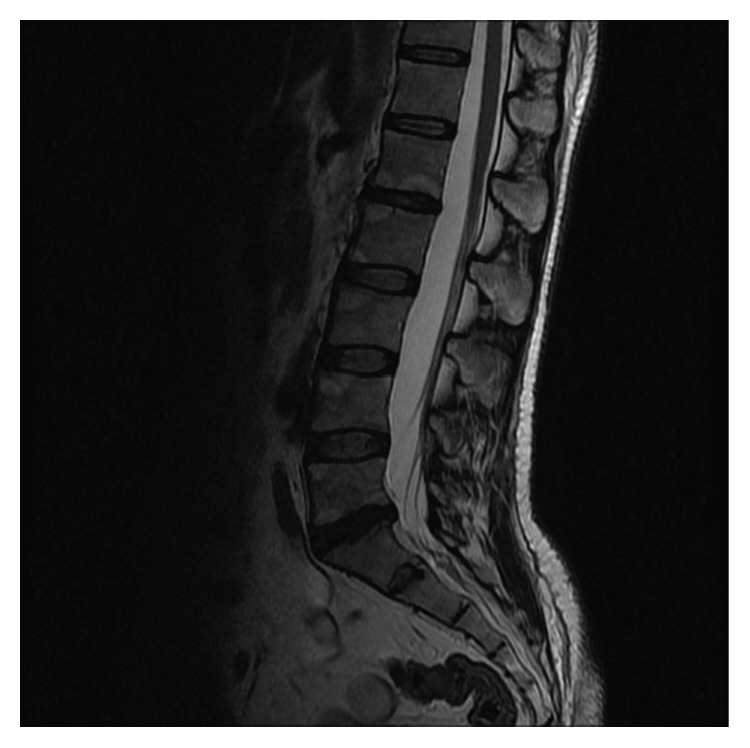
T2 sagittal MRI scan of the lumbar spine revealing Modic type 2 degeneration.

A 30-year-old female (the first patient's sister) was admitted to the outpatient clinic with inflammatory back pain complaints for 4 years, morning stiffness lasting 2 hours, a history of transient heel pain, and no history of arthritis. In addition, her examination did not indicate any actual enthesitis despite family history. Complete blood count demonstrated 6,700 cells/*µ*L WBC (N: 4,100–11,000), 4,510 cells/*µ*L neutrophils (N: 2,000–8,000), 12.7 g/dL Hgb (N: 11–18), 38.5% HCT (N: 35–55), and 208,000/*µ*L PLT (N: 150,000–400,000). She had levels of CRP 5.56 mg/dL (N ≤ 3.48), ESR 28 mm/h (N: 0–20), sCR 0.54 mg/dL (N: 0.67–1.17), AST 19 U/L (N: 0–50), and ALT 11 U/L (N: 0–50). Total urinalysis findings showed 1.020 density (N: 1.015–1.020), 6.0 pH (N: 5.0–7.0), 10 erythrocytes, and 42 leukocytes. The Rose Bengal test, HBs-Ag, anti-HCV, anti-HIV, and HLA-B27 were negative. A plain anteroposterior radiograph of the patient's pelvis showed grade 3 sacroiliitis on the right side and grade 2 sacroiliitis on the left side (Figure 4). A T2 fat-suppressed sacroiliac joint MRI demonstrated widespread edematous T2 signal augmentation on the sacral sides of the bilateral sacroiliac joints, indicating acute sacroiliitis and bilateral sclerosis (Figure 5). Lumbar MRI revealed Modic type 2 degeneration on the anterior aspects of L1, L2, and L3 vertebrae, indicating old Romanus lesions, multiple cysts, and enlarged kidneys (Figures 6 and 7). She was diagnosed with axial SpA according to ASAS 2009 axial spondyloarthritis classification criteria [13]. The clinical features, laboratory findings, and imaging results of the patient are shown in Table 1. She had a BASDAI of 5.8 and a BASFI of 5.3. After consulting with a nephrologist, she was also diagnosed with ADPKD and started on acemetacin 90 mg daily under strict monitoring. Three weeks after initial treatment, her results were WBC 7,100 cells/*µ*L, neutrophils 4,780 cells/*µ*L, CRP 14.6 mg/dL, ESR 30 mm/h, sCR 0.58 mg/dL, AST 11.4 U/L, and ALT 10 U/L; with the exception of 15 erythrocytes, total urinalysis was within normal limits. Morning stiffness duration improved to 30 minutes, with a BASDAI of 5 and a BASFI of 4.7. Her medication was changed to dexketoprofen trometamol 50 mg/day. One month later, WBC was 8,300 cells/*µ*L, neutrophils 5,330 cells/*µ*L, CRP 3.51 mg/L, ESR 24 mm/h, serum uric acid 5.06 mg/dL, sCR 0.58 mg/dL, AST 10 U/L, and ALT 12 U/L. BASDAI was 5.1, BASFI was 4.2, and morning stiffness duration was 20 minutes. She was followed up for 1 year and did not have any acute episodes, but experienced apparent bacteriuria with symptoms and required antibiotics twice. Her final results were WBC 8,700 cells/*µ*L, neutrophils 6,150 cells/*µ*L, Hgb 12 g/dL, HCT 37.8%, PLT 196,000/*µ*L, CRP 1.26 mg/dL, ESR 15 mm/h, sCR 0.72 mg/dL, serum uric acid 6.6 mg/dL, AST 10 U/L, and ALT 12 U/L, with a spot urine protein-to-creatinine ratio of 0.09. Final total urinalysis showed 5 erythrocytes, 6 leukocytes, and 17 squamous epithelium. BASDAI was 4.3 and BASFI was 4, and the patient had morning stiffness for 1 hour, making her a candidate for an anti-TNF agent.

## 3. Discussion

Renal involvement among AS patients was reported as high as 13.3% [10–12]. Therefore, patients with AS should be regularly monitored for renal complications. Ironically, NSAIDs are recommended as first-choice drugs for axial SpA, and NSAID-caused tubulointerstitial nephritis is another important cause of renal complications [8, 14]. Also, a recent study demonstrated that frequent NSAID use may cause 2- to 3-fold elevation of kidney injury molecule 1 (KIM1), cystatin C (Cys-C), and neutrophil gelatinase-associated lipocalin (NGAL) in urine and serum due to acute kidney injury; serum levels of these molecules return to normal 12 weeks later following drug cessation [11]. However, end-stage renal disease as a result of long-term NSAIDs is rare without preexisting kidney dysfunction [11, 15]. For patients with impaired renal function, prostaglandin production mediated by cyclooxygenase 1 (COX-1) and COX-2, which are inhibited by NSAIDs, has a major compensatory effect in sustaining renal hemodynamic function [16].

Screening the extra-articular manifestations in axial SpA patients may influence treatment decisions [17]. Physicians may vacillate between types of NSAID used to alleviate disease activity and preserve kidney function. Patients with ADPKD generally progress to end-stage renal disease by 60 years of age; 70% of these patients require renal transplantation by 70 years of age [18]. Delaying the progressive kidney function loss or end-stage renal disease significantly improves quality of life in these patients [4]. In such cases, anti-TNF-*α* treatment is indicated for conserving renal function. It is not reported to be contraindicated in renal impairment cases, and TNF-*α* induces glomerular inflammation and permeability [19]. Lee et al. [9] reported significant reduction in proteinuria due to amyloidosis from 3,702 mg/day to 200 mg/day in an AS patient treated with etanercept for 12 months. They reported an AS patient with accompanying IgA nephropathy in whom CRP and BASDAI levels were normalized using infliximab although proteinuria was unalleviated. In the same study, another AS patient with IgA nephropathy, who was treated with adalimumab, showed proteinuria alleviation. They concluded that initial serum creatinine levels may be important in predicting anti-TNF-*α* treatment response [9].

Jacquet et al. [20] reported an AS patient with normal renal function who developed microscopic hematuria and proteinuria after 2-year initial anti-TNF-*α* treatment and was treated with infliximab. The authors determined that hematuria remained at 3 years and proteinuria increased to 1.75 g/day. Renal biopsy demonstrated IgA nephropathy. Anti-TNF-*α* treatment induces a shift from T-helper type-1 pattern (e.g., interleukin 1 (IL-1), TNF, and interferon gamma (IF*γ*)) to T-helper type-2 pattern (e.g., IL-4, IL-5, IL-6, IL-10, and IL-13), hence promoting antibody-mediated immunity; this may lead to IgA-mediated renal involvement [20].

Lee et al. [21] monitored a patient with secondary amyloidosis due to AS. Following etanercept treatment for 17 months, amyloid deposits showed slightly regressed histopathology even though proteinuria recovered. Anti-TNF treatment probably alleviates proteinuria by reducing serum amyloid A levels but is insufficient for resolving amyloid deposits [21, 22].

To our knowledge, this is the first case reporting an association between axial SpA and ADPKD. This cooccurrence may be coincidental, or an undiscovered alternate gene or pathway may be responsible for this association considering the HLA-B27 negativity of both patients. Alterations in PC1 and PC2 function result in changes in intracellular calcium and cyclic adenosine monophosphate (cAMP) levels and subsequent mechanistic target of rapamycin (mTOR) pathway alterations [5]. mTOR-controlled metabolic pathways are likely to shape the repertoire of both adaptive and innate inflammatory cells in AS, making the mTOR pathway a possible cause of increased inflammation and AS [23].

In conclusion, although dependency of axial SpA and ADPKD is not well known, this study shows a possible link between these two diseases. Practitioners should consider renal involvement in axial SpA patients and make treatment decisions according to renal complications.

## Figures and Tables

**Table 1:** Clinical features, initial test results, and imaging results of patients.

	Case 1	Case 2
*Age*	37	30
*Gender*	Male	Female
*Symptoms*		
	Inflammatory back pain and 20 minutes of morning stiffness	Inflammatory back pain, 2 hours of morning stiffness, and transient heel pain history
*Laboratory findings*		
White blood cells (WBCs) (N: 4,100–11,000 cells/*µ*L)	6,700	6,700
Neutrophils (N: 2,000–8,000 cells/*µ*L)	4,240	4,510
Hemoglobin (Hgb) (N: 11–18 g/dL)	13.02	12.7
Hematocrit (HCT) (N: 35–55%)	41.3	38.5
Platelets (PLTs) (N: 150,000–400,000 cells/*µ*L)	146,000	208,000
C-reactive protein (CRP) (N ≤ 3.48 mg/dL)	6.71	5.56
Erythrocyte sedimentation rate (ESR) (N: 0–20 mm/h)	8	28
Serum creatinine (sCR) (N: 0.67–1.17 mg/dL)	0.81	0.54
Aspartate transaminase (AST) (N: 0–50 U/L)	16.9	19
Alanine transaminase (ALT) (N: 0–50 U/L)	19	11
Total urinalysis findings	1.011 density (N: 1.015–1.020), and other parameters were normal	1.020 density (N: 1.015–1.020), 10 erythrocytes, and 42 leukocytes
The Rose Bengal test	Negative	Negative
Hepatitis B surface antigen (HBs-Ag)	Negative	Negative
Anti-hepatitis C virus (HCV)	Negative	Negative
Anti-human immunodeficiency virus (HIV)	Negative	Negative
Human leukocyte antigen- (HLA-) B27	Negative	Negative
*Imaging*		
	T1-weighted, fat-suppressed, gadolinium-enhanced MRI scan showed acute sacroiliitis	Pelvis anteroposterior radiograph showed right grade 3 sacroiliitis and left grade 2 sacroiliitis
	MRI scan indicated old Romanus lesions and polycystic kidneys	MRI scan indicated old Romanus lesions and polycystic kidneys
